# Impact of Point-of-Sale Tobacco Display Bans in Thailand: Findings from the International Tobacco Control (ITC) Southeast Asia Survey

**DOI:** 10.3390/ijerph120809508

**Published:** 2015-08-13

**Authors:** Lin Li, Ron Borland, Hua-Hie Yong, Buppha Sirirassamee, Stephen Hamann, Maizurah Omar, Anne C.K. Quah

**Affiliations:** 1 Nigel Gray Fellowship Group, Cancer Council Victoria, 615 St. Kilda Road, Melbourne, Victoria 3004, Australia; E-Mails: Ron.Borland@cancervic.org.au (R.B.); Hua.Yong@cancervic.org.au (H.-H.Y.); 2 Institute for Population and Social Research, Mahidol University Salaya, Phutthamonthon, Nakhon Pathom 73170, Thailand; E-Mail: buppha.sir@mahidol.ac.th; 3 Tobacco Control Research and Knowledge Management Center, Mahidol University, Bangkok 10400, Thailand; E-Mail: slhamann@gmail.com; 4 National Poison Center, University Sains Malaysia, 11800 Minden, Penang, Malaysia; E-Mail: maizurah@usm.my; 5 Department of Psychology, University of Waterloo, 200 University Avenue West, Waterloo, ON N2L 3G1, Canada; E-Mail: ackquah@uwaterloo.ca

**Keywords:** tobacco products, advertising and promotion, regulations, point-of-sale, Thailand, Malaysia

## Abstract

In September 2005 Thailand became the first Asian country to implement a complete ban on the display of cigarettes and other tobacco products at point-of-sale (POS). This paper examined the impact of the POS tobacco display ban in Thailand, with Malaysia (which did not impose bans) serving as a comparison. The data came from the International Tobacco Control Southeast Asia Survey (2005–2011), a prospective cohort survey designed to evaluate the psychosocial and behavioral impacts of tobacco control policies. Main measures included smokers’ reported awareness of tobacco displays and advertising at POS. At the first post-ban survey wave over 90% of smokers in Thailand were aware of the display ban policy and supported it, and about three quarters thought the ban was effective. Noticing tobacco displays in stores was lowest (16.9%) at the first post-ban survey wave, but increased at later survey waves; however, the levels were consistently lower than those in Malaysia. Similarly, exposure to POS tobacco advertising was lower in Thailand. The display ban has reduced exposure to tobacco marketing at POS. The trend toward increased noticing is likely at least in part due to some increase in violations of the display bans and/or strategies to circumvent them.

## 1. Introduction

Tobacco advertising, promotion, and sponsorship (TAPS) are associated with smoking initiation and use [1,2]. World Health Organization Framework Convention on Tobacco Control (FCTC) recognizes that meaningful tobacco control must include the elimination of all forms of TAPS [3]. An increasing number of jurisdictions have prohibited TAPS in traditional media outlets such as broadcast, print, and outdoor billboards. As a result, the tobacco industry has increasingly turned to point-of-sale (POS) tobacco displays and promotion as an important means of marketing their products [1,4,5]. Increasing evidence shows that the widespread presence of cigarette displays at the POS increases the likelihood that youth will start smoking [1,4,5,6,7], and stimulate impulse purchasing and use among current smokers [4,7,8,9,10,11,12].

To address this serious problem, some jurisdictions have implemented POS tobacco product display bans/restrictions, combined with other tobacco control efforts [12,13]. For example, as a part of comprehensive tobacco control measures, POS display bans have been in place for some time in Iceland (since 2001) and Canada (with gradual implementation) [14], and there has been a decrease in youth and/or adult smoking prevalence [15,16]. Although the independent effects of the display bans are difficult to be separated from other measures, these bans may have contributed to the reductions in these countries [15,16]. Li and colleagues examined the impact of POS display bans in Australia and Canada, in relation to the United Kingdom and the United States where there were no such restrictions between 2006 and 2010, and found that implementing POS display bans reduces smokers’ exposure to tobacco marketing and lowers their reported impulse purchasing of cigarettes [12].

**Figure 1:**
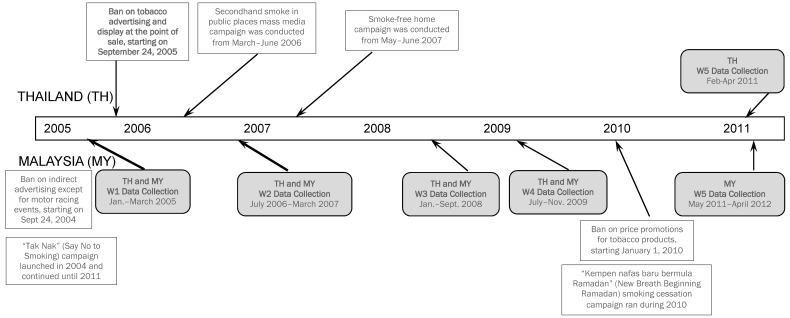
Timeline of national tobacco advertising, promotion, and sponsorship policies in relation to data collection at each survey wave in Thailand and Malaysia.

The policy impact of POS display bans in developing countries has not been systematically studied and this study sought to fill this gap using data from the International Tobacco Control Southeast Asia (ITC SEA) smoker survey collected between 2005 and 2011 in Thailand and Malaysia. Thailand is a leader in tobacco control in Asia. It ratified the FCTC in 2004, and has been compliant with most requirements of the FCTC [17]. Its substantial tobacco control efforts include adopting taxation/price measures, and enhancing health warnings on cigarette packaging (requiring graphic warning labels from March 2005 (after the baseline survey of this study)) [17,18]. Thailand conducted its first mass media anti-smoking campaign in late 2005 (between Waves 1 and 2 of our data collection). In addition, Thailand has notable legislations on TAPS restrictions. Its Tobacco Products Control Act 1992 comprehensively banned advertising and promotion and made most forms of promotional activities illegal [19]. In September 2005 (between the first two survey waves, see Figure 1 below), Thailand became the first Asian country to implement a complete ban on the displays of cigarettes and other tobacco products at the POS [14,20]. 

This initiative was not expected, and unfortunately we did not have measures of awareness in place before the ban. This limits what can be achieved, but given the importance of the issue, we believe the limited evaluation we have been able to conduct can provide new and important information. Some early data show that the reported awareness of tobacco advertising and promotion in Thailand was low [21,22], but to date there has been no systematic effort to evaluate the overall impact of the 2005 display bans. 

Malaysia and Thailand are neighbors. Malaysia is relatively more economically advanced and urbanized compared to Thailand. For the most part of the study period, Malaysia had comparatively fewer tobacco control policies and measures. Malaysia had a small, general, text-only warning on one side of cigarette packs throughout the first three waves of our ITC SEA data collection and only started to implement pictorial warning labels from January 2009 (between Waves 3 and 4 of data collection) [23]. Malaysia also introduced some price/tax measures during the study period, and it launched its national campaign earlier than Thailand, from 2004 (but the implementation in Malaysia was considerably weakened by early 2005 and was dormant during much of the study period). 

Important for this study, Malaysia had not adopted any display bans/restrictions on POS advertising or other strong measures during the study period except that it introduced a ban on price promotions for tobacco products in early 2010. Note: In 2004, Malaysia introduced regulations that included a ban on tobacco advertising and promotion and smoke-free policies [23]. Research from other countries shows that implementing tobacco marketing bans helps reduce exposure to advertising and promotion [21,22]. 

The specific aim of this study is to examine the impact of the POS tobacco display bans in Thailand and the longer term bans on POS advertising, with Malaysia serving as a comparison country.

## 2. Methods

### 2.1. Data Source and Participants

The data came from the ITC SEA smoker survey, a prospective cohort survey designed to evaluate the psychosocial and behavioral impacts of tobacco control policies in Malaysia and Thailand. A detailed description of the sampling and study design of the survey has been reported elsewhere [21,24]. Briefly, the ITC SEA adult smoker survey employs a multistage clustering sampling procedure. Participants were recruited from adults who had smoked at least 100 cigarettes in their lifetime and smoked at least weekly at the time of recruitment. All participants were surveyed using standardized questionnaire (in local languages). All participants in Thailand were surveyed via face-to-face interviews. In Malaysia, Wave 1 survey was conducted via face-to-face interviews; for Waves 2 and 3, both face-to-face and telephone interviews were used; Wave 4 interviews were conducted by telephone, and Wave 5 by the Computer-Assisted Telephone Interview system [24,25].

All smokers gave their informed consent for inclusion before they participated in the study. The study was conducted in accordance with the Declaration of Helsinki, and the study protocol was approved by the institutional review/research ethics boards from the University of Waterloo (Canada, Project No. 15468 and 11762), Cancer Council Victoria (Australia, Project No. HREC 0424), Mahidol University (Thailand), and Universiti Sains Malaysia (Malaysia). 

The sample size per country was initially around 2000 at each survey wave, with replenishment sampling from the same sampling frame used to maintain sample size across waves. The analyses reported in this paper are restricted to current smokers at the time of surveying. The number of smokers in each country at each survey wave/year (Waves 1 to 5, from 2005 to 2011) and their characteristics are summarized in Table 1. 

### 2.2. Measures

#### 2.2.1. Sociodemographics and Smoking-Related Variables 

Sociodemographic variables included sex (male, female), age (18–24, 25–39, 40–54, 55 and older), ethnic group (majority *vs.* minority groups), region type (urban, rural), and education and income (low, moderate, high) [26]. Smoking-related measures included cigarettes per day (CPD, “1–10”, “11–20”, “21–30” and “31+”); intention to quit (planning to quit “within the next month”, “within the next six months”, “beyond six months” and “not planning to quit”); and self-efficacy for quitting successfully (“not at all sure”, “somewhat sure/don’t know”, “very sure” and “extremely sure”). 

#### 2.2.2. Tobacco Advertising and Displays at Point-of-Sale

At each survey wave, participants were asked if in the last six months they had noticed cigarettes or tobacco products being advertised (1) on store windows or inside stores where they buy tobacco; and (2) on or around street vendors. From Wave 2 onward, they were also asked (3) if they had seen cigarette displays in shops in the last month. Response options were “yes”, “no” and “don’t know” with the last two categories being coded as “not exposed”.

In addition, at the Wave 2 Survey smokers in both countries were asked about their support for a complete ban on displays and advertising in shops; smokers in Thailand were also asked about their awareness of the display ban policy and its effectiveness. 

### 2.3. Data Analysis

Group differences for categorical variables were examined using chi-square tests. Taking into consideration the correlated nature of the data within participants across survey waves, we used the Generalized Estimating Equations (GEE) modeling to compute parameter estimates and examine if there were any differences across waves in the exposure to POS tobacco marketing. In such modeling, each exposure variable was treated as a dependent variable (binary), with “survey wave” as an independent variable, plus controlling for demographics. An α level of *p* < 0.05 was used for all statistical tests. Data analyses were conducted using Stata Version 12.1.

## 3. Results

### 3.1. Sociodemographic and Smoking-Related Characteristics

Table 1 summarizes the sociodemographic and smoking-related characteristics of the sample. In both countries, the vast majority (over 90%) of participants were male. In Thailand, the participants were overwhelmingly of the Thai ethnic group (almost 99%). Among the Malaysians, about 79% were Malays. Compared to their Thai counterparts, Malaysian smokers were more likely to be from urban regions, with moderate or high education, and to be younger (64% of Malaysian smokers were younger than 40 years). Compared to Malaysia, a bigger proportion of smokers in Thailand reported smoking 1–10 cigarettes per day (54.7%:48.3%), having no intention to quit (65.3%:39.1%), and no self-efficacy for quitting successfully (39.3%:21.6%) (all *p* values < 0.001). 

### 3.2. Noticing Cigarette Displays over Time 

**Figure 2:**
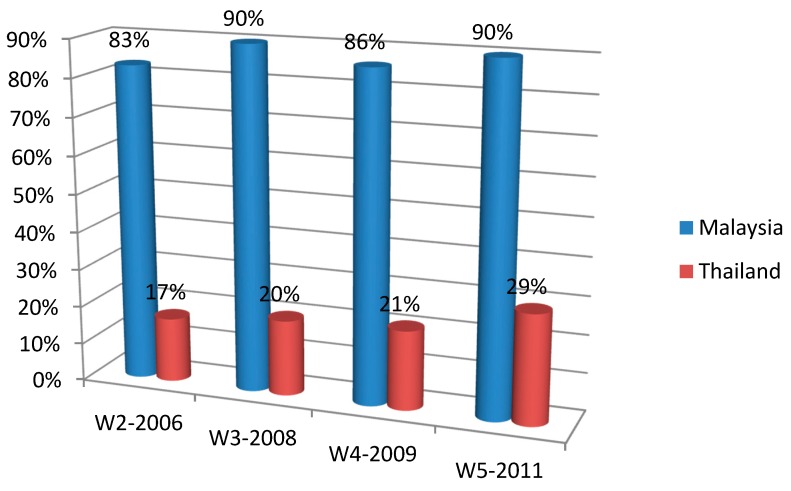
Reported exposure to cigarette displays in shops in Malaysia and Thailand.

As can be seen from Table 2 and Figure 2, overall the proportions of smokers reporting having noticed displays in stores in Thailand were lowest (about 17%) in 2006 shortly after the ban was enforced, but increased at later survey waves (overall wave effect testing *p* < 0.001, GEE modeling results: odds ratios range from 1.21–1.80, Table 2); however, the levels were consistently lower than those in Malaysia (where over 82% noticed displays across the waves; country differences were significant at all waves, all *p* values < 0.001). In both countries, younger smokers were overall more likely than older ones to notice displays (Table 2). In Malaysia, urban smokers were overall more likely to notice displays than their rural counterparts; and this is also the case for Thailand at Wave 5. In Thailand, smokers in Bangkok were less likely to notice displays than smokers in other regions at Wave 3 but more likely to notice at Wave 5.

**Table 1:** Sample characteristics, by country.

Characteristics	Malaysia	Thailand	Total	Country Differences~
No. of current smokers at each wave **^**				
Wave 1 (in early 2005)	2004	2000	2004	
Wave 2 (2006)	1550	1866	3416	
Wave 3 (2008)	1846	2163	4009	
Wave 4 (2009)	1888	1907	3795	
Wave 5 (2011)	1773	1706	3479	
Sex (% male, out of total unique individuals #: for Malaysia *n* = 4787; for Thailand *n* = 3584)	97.3	91.2	94.7	**
Identified minority group (%)	21.2	1.4	12.9	***
Urban/rural region (% urban)	65.4	42.4	55.6	***
Age at recruitment (%) **#**				***
18–24	31.1	8.1	21.2	***
25–39	32.9	26.1	29.9	
40–54	24.1	38.8	30.5	
55+	11.9	27.1	18.5	
Education at recruitment (%)				***
Low	15.2	68.8	38.5	
Moderate	54.9	22.9	41.1	
High	29.8	8.3	20.5	
Income at recruitment (%)				
Low	28.1	25.7	26.1	***
Moderate	34.2	30.9	32.8	
High	29.4	39.7	35.3	
No information	8.4	3.8	5.8	
Cigarettes per day at recruitment (%)				***
1–10	48.3	54.7	51.1	
11–20	45.8	38.3	42.6	
21–30	3.5	4.6	4.0	
31+	2.4	2.5	2.4	
Intention to quit at recruitment (%)				***
No intention/can’t say	39.1	65.3	50.4	
Beyond 6 months	47.3	16.1	33.8	
Within next 6 months	8.2	12.4	10.0	
Within next month	5.4	6.2	5.8	
Self-efficacy at recruitment (%)				***
Not at all sure	21.6	39.3	29.3	
Somewhat sure/don’t know	53.6	33.8	45.1	
Very sure	19.5	17.7	18.7	
Extremely sure	5.3	9.2	7.0	

**^** For the numbers of new recruits at each survey wave please refer to the ITC-SEA technical report (http://www.itcproject.org/countries/thailand). **#** For all unique individuals who were presented in at least one wave of the surveys (from Wave 1 to Wave 5), and this applies to the other variables in the table. For some variables the numbers of cases were fewer than the total unique cases, due to some “don’t know” and “missing” cases. ~chi square test results. ** Significant at *p* < 0.01; *** at *p* < 0.001.

**Table 2:** Current smokers’ reported exposure to POS tobacco displays and advertising.

Exposure	Malaysia					Thailand				
	W1 *n* = 2004 ^	W2 *n* = 1550	W3 *n* = 1846	W4 *n* = 1888	W5 *n* = 1773	W1 *n* = 2000	W2 *n* = 1866	W3 *n* = 2163	W4 *n* = 1907	W5 *n* = 1706
Noticed cigarette displays in shops (% yes)	N.A	82.7	89.6	85.8	90.3	N.A	16.9	20.3	20.5	29.1
Wave difference: OR !		Ref.	1.72 ***	1.04	1.44 ***		Ref.	1.22 **	1.21 **	1.80 ***
- Younger:older **#**		86.6:79.4 ***	93.3:85.9 ***	87.1:83.6	92.9:81.4 ***		21.1:15.2 **	24.6:18.3 **	23.9:18.9 *	35.4:25.9 ***
- Urban:rural **#**		85.9:77.8 ***	91.8:86.2 ***	85.9:86	91.1:88.8		18.3:16.2	18.6:21.1	19.8:21.1	31.9:26.6 *
- Malaysia only, f2f:phone **##**		83.1:82.1	86.1:92.6 ***							
- Thailand only, 1Bangkok:2other urban:3rural							19.4:17.9:16.2	13.6:20.6:21.3 *	21.4:19.1:21.0	37.2:28.7:26.6 **
Noticed tob ads in stores (% yes)	55.4	58.5	27.9	26.7	45.1	3.8	7.6	2.7	10.8	9.2
Wave difference: OR	Ref.	1.06	0.30 ***	0.28 ***	0.62 ***	Ref.	2.09 ***	0.77	3.22 ***	2.48 ***
- Younger:older	59.2:51.7 **	59.4:57.2	24.1:31.5 **	26.1:28	47.1:39.3 **	4.1:3.6	7.8:7.6	3.1:2.6	11.7:10.4	9.8:8.9
- Urban:rural	51:62.6 ***	58:59.3	22.2:36.9 ***	25.3:28.9	43.4:47.9	4.6:3.5	6.3:8.3 *	1.4:3.4 **	8.9:12.4 *	8.6:9.7
- Malaysia only, f2f *:Phone		59.9:55.8	47.9:11.3 ***							
- Thailand only, 1Bangkok:2other urban:3rural						7.2:3.3:3.4 *	6.4:6.3:8.3	0:2.1:3.4 *	7.4:9.6:12.4 *	10.2:7.7:9.7
Noticed tob ads around street vendors (% yes)	47.2	43.6	23.2	16.4	29.1	7.1	9.6	4.2	7.8	10.4
Wave difference: OR	Ref.	0.83 **	0.34 ***	0.22 ***	0.47 ***	Ref.	1.40 **	0.67 **	1.24	1.58 ***
- Younger:older	51.2:43.3 **	42.7:43.6	19.7:26.8 ***	15.7:18.2	29.4:28.1	4.9:8 *	7.4:10.6 *	2.7:4.8 *	6.6:8.4	10.3:10.4
- Urban:rural	44.8:51.2 **	44.2:42.7	20.5:27.6 *	15.7:17.5	28.5:30.1	2.3:9.1 ***	5.1:12.1 ***	1.2:5.7 ***	5.4:9.8 ***	8.8:11.8 *
- Malaysia only, f2f:phone		42.9:44.9	34.9:13.5 ***							
- Thailand only, 1Bangkok:2other urban:3rural						1.4:2.8:9.1 ***	4.3:5.4:12.1 ***	0:1.7:5.7 ***	5.2:5.4:9.8 **	12.3:6.7:11.8 **

Notes: **^** For some analyses the numbers of cases were fewer than the total, due to some “refused” and “missing” cases. “W” stands for “Wave of survey”. N.A: Not available (not asked at the specific survey wave). ! OR: Odds ratio; generalized estimating equations modelling results, controlled for demographics. Ref: reference value. * Significant at *p* < 0.05; ** at *p* < 0.01; *** at *p* < 0.001. **#** Younger: 18–39 years old; older: 40+ years; chi square tests were used. **##** “f2f”: Face to face interview mode; “phone”: telephone interview. All surveys in Thailand were conducted through face to face interviews. In Malaysia, mix methods were used for Waves 2 and 3; Wave 1 surveys were all conducted by face to face interviews; Wave 4 all by phone and Wave 5 all by computer assisted telephone interview.

### 3.3. Noticing Tobacco Advertising at POS over Time

For noticing tobacco advertising at POS, smokers in Thailand consistently reported lower levels (less than 11% in both stores and around street vendors) than those reported in Malaysia (at least 26% in stores and higher than 16% around street vendors; country differences were significant at all waves). In Thailand, the noticing levels increased somewhat in later waves, especially from Waves 3 to 4. 

Overall, smokers in rural areas in Thailand were more likely to notice advertising at POS than their urban counterparts (except at Wave 1, Table 2), and a similar pattern was also found in Malaysia. In Thailand, compared to the younger smokers, older smokers were more likely to notice advertising around vendors, especially at early waves (Table 2). The trend in Malaysia was not consistent. 

### 3.4. Additional Results on POS Tobacco Display Bans (2006 Survey Only)

At the first post-ban survey (in 2006), smokers in Thailand were asked if they were aware of the display ban policy, and 91% of smokers said they were aware of it; when asked about the effectiveness of the display ban, 77% thought the ban was effective (older smokers (40+) were more likely to say the ban was effective than their younger counterparts (81%:67%, *p* < 0.001); these additional results are not reported in Table 2). At the same survey, smokers in both countries were asked if they supported complete ban on displays, and 92% of Thai smokers and 79% of Malaysian smokers said yes. Similar proportions of smokers (92% in Thailand and 81% in Malaysia) said they supported a complete ban on tobacco ads in shops. 

## 4. Discussion

The POS tobacco display bans appeared to have reduced but not eliminated exposure to cigarette pack displays at POS in Thailand, especially in the first year after the policy was introduced. This can be inferred from the large differences found between reports in Malaysia where displays are allowed, and the markedly smaller rates in Thailand. Unfortunately, we do not have pre-implementation data on noticing displays in shops from Thailand, so it is unclear how much of the difference is due to the removal of the products and how much to the removal of advertising which occurred in 2004. Support for display bans was high (over 90% in Thailand), and about three quarters of smokers thought the ban was effective. 

Ideally it would be useful to utilize data to show how changes in exposure to cigarette displays/marketing at POS further influenced smokers’ behavior. Unfortunately, in the ITC SEA survey smokers’ cigarette purchasing behavior questions were not asked, therefore we could not conduct behavioral analysis on this. However, we did additional analysis on how smokers’ cigarette consumption changed from Wave 1 in 2005 (pre-ban) to Wave 2 in 2006 (post-ban) (Note: This was not reported above in the main results), and we found that among Thai smokers the mean number of cigarettes smoked per day significantly reduced from 13.2 in 2005 to 12.2 in 2006 (*p* < 0.001), whereas in Malaysia no significant reduction was observed during the same period (the means were about 13.7 at both survey Waves). We are aware that it could be inappropriate or premature to say that it was the introduction of the display ban or the change in exposure to POS cigarette display/marketing that caused the reduction in cigarette consumption among Thai smokers, because there might be many other factors that could contribute to the change in consumption reduction, and the time of occurrence of exposure and cigarette consumption reduction was not certain, so here any claim on causal relation between these two might be problematic. This is one of the limitations of this study, and other limitations will be discussed later. In another ITC study that used the ITC Four Country Survey data, smokers were asked if tobacco displays at POS made them buy unplanned cigarettes, and the results show that POS tobacco display bans in Australia and Canada were associated with lowered reports of unplanned purchasing [12]. 

While the bans have overall been effective in reducing exposure in Thailand, the rising trend of noticing both displays and advertising is a cause for concern. There are a number of possible reasons for the observed rises: first, it could represent a change in reference point, such that rare events are now noticed whereas previously, the shift from ubiquitous exposure to rare meant they were ignored; second, this could be due to an increased interest in smokers to see what is available; third, it could represent increased violations of the law; and finally we need to take into consideration the fact that smokers often buy tobacco products, and when doing so, storage compartments have to be opened; depending on how they are positioned and the products stored, this may provide an easy opportunity to view the contents. 

Because smokers are more likely to see displays when opened, caution should be exercised in generalizing from these findings to what non-smokers might see. However, what they see is dependent on what happens when the cigarette storage door is opened. If it is under the counter, chances of seeing tobacco displays would be minimal. If it is in cupboards behind the counter and the packs are organized to be prominent when the door is opened, the chances of seeing the entire display are far greater. A further reason for not generalizing our findings to non-smokers is that in some other places where it has been studied, non-smokers report less awareness of advertising, suggesting they are less cued to it [21,22].

With this context in mind, we now turn to whether the observed effects could be some form of observer effect, or are more likely to reflect increased violations of the laws. To do this, we turn to an analysis of differences in awareness within waves. Some differences could be due to different kinds of stores visited, the most obvious example being between rural and urban participants, but this also applies to other demographics: young people frequent different kinds of stores than older people, and so forth. We observed such effects: younger smokers tended to be more likely to notice displays and less likely to think the display ban was effective; there is also a regional disparity in noticing. However, this could also be a differential sensitivity effect, in that young people are generally more sensitive to advertising in general [27], while rural people are exposed to much less advertising and so a small amount of it may stand out. That displays were noticed more by urban respondents at Wave 5 in Thailand suggests the exposure was likely real as the marketing must have at least stood out from all other types of marketing. However, we cannot discount the possibility that some non-smoking promotion was assumed to be for tobacco, even though this seems unlikely. This suggests greater problems with the display ban in urban areas. However, it may be that in urban areas there is greater likelihood that the turnover makes it more economical to put in place containers that afford high visibility to products when the door is opened. 

In our view, the evidence here coupled with evidence from elsewhere points to a reduction in effective enforcement of the bans. Although Thailand has had a ban on all direct and indirect tobacco advertising and promotion, there are some loopholes in enforcement and implementation, especially when compared to countries such as Canada, where a compliance rate (of surveyed retail outlets) of as high as 99.8% was reported [28]. Findings from a recent study conducted in Thailand indicate that 21% of cigarette retail shops provided credit to some regular adolescent smokers to buy cigarettes and allowed them to take cigarettes by themselves from POS [29]. In our ITC SEA project we do not have cigarette retailer compliance data, and we do not know if what we have found is due to reduced compliance by retailers alone or is supported by more systematic activity of tobacco companies. The cupboards at POS may have also been kept open more often, both intentionally and unintentionally. Some level of noticing this has been informally reported by Thailand-based authors, both based on anecdotal reports from others and their own observations when in the field. 

As both reporting advertising and noticing displays have increased, we wondered if both effects could be due to taking more notice of displays, as seeing packs at POS is one form of advertising. We checked our data and found there is some positive correlation between noticing displays and noticing store advertising, but it is only low and no higher than in Malaysia, where advertising was still allowed (correlations in Thailand ranged from 0.10 to 0.26; in Malaysia, they ranged from 0.07 to 0.17). Further, the different patterns of change by some sociodemographics make this unlikely to be a major reason. If so, then there may in fact be more direct violations of the advertising bans.

This analysis is consistent with the fact that the tobacco industry is finding innovative ways to advertise and promote, perhaps some within the law, and others possibly illegal. We are concerned that organized forces within the industry may be pushing the envelope in Thailand, along perhaps with more localized lack of vigilance about complying with the laws. Without strong enforcement, things can backslide quickly. 

Overall, higher levels of exposure were reported by Malaysian smokers than their Thai counterparts. There was also some rather large wave–to-wave variability that we are unable to fully explain. Some of the differences may be mode effects as we shifted from face-to-face to phone surveying in Malaysia. The levels of noticing advertising declined considerably at Waves 3 and 4, and at Wave 3, there were contrasting differences between face-to-face interviews and those via telephone (e.g., 47.9% *vs.* 11.3% exposure to advertising in stores). While some of this may be due to differences in the characteristics of participants, some is likely due to the mode of survey [30]. Therefore, caution needs to be exercised when comparing the absolute levels of changes over time and between countries.

In spite of some limitations, we have been able to show that the tobacco display ban in Thailand has reduced (but not eliminated) exposure to tobacco displays at POS. The higher level of noticing POS displays than advertising suggests displays are generally more salient to smokers. Findings are consistent with those from Western countries. While we cannot be sure, the trends to increased noticing are likely to be at least in part due to some increase in violations of the bans and/or strategies to circumvent them. Thai authorities might usefully review enforcement procedures and review the legislation to make it harder for tobacco displays to be used when storage compartments are opened.

## 5. Conclusions

The tobacco display ban in Thailand appeared to have reduced but not eliminated exposure to tobacco marketing at point-of-sale. There was a rising trend of noticing both tobacco displays and advertising. The findings highlight the need to reinforce enforcement of the bans.
